# Hypersensitivity Reaction to Metal: A Bibliometric Study

**DOI:** 10.1177/23779608221132164

**Published:** 2022-10-11

**Authors:** Tassia Teles Santana de Macedo, Itana Lúcia Azevêdo de Jesus, Wilton Nascimento Figueredo, Dzifa Dordunoo

**Affiliations:** 1 Escola Bahiana de Medicina e Saúde Pública—Enfermagem, Salvador, Bahia, Brazil; 2 Universidade Estadual de Feira de Santana, Departamento de Saúde, Feira de Santana, Bahia, Brazil; 3 8205 University of Victoria, School of Nursing, Victoria, BC, Canada

**Keywords:** adverse effects, allergy, hypersensitivity, metals, bibliometric literature review

## Abstract

**Background:** To delineate the scientific publications on metal hypersensitivity. **Methods:** Scopus database from 1946 to 2020, written in English, Spanish, or Portuguese. This is a bibliometric study, with a descriptive and quantitative approach. For data analysis, we used RStudio® and VOSviewer® and bibliometric packages—bibliometrix and biblioshiny. **Results:** Of the 804 articles retrieved, most of the publications come from European, Asian, and American countries, with Germany, Japan, and United States leading. Published articles and keywords refer to orthopedic, dermatological, and orthodontic specialties. **Conclusion:** Scientific production is scarce with slight oscillations in the studied period, authored predominantly by researchers in North America and Europe. Articles were mostly published in scientific journals in the fields of dermatology, dentistry, and orthopedics, which indicated the need for greater investments in the research development on the topic.

## Introduction

Hypersensitivity to metal is a Type IV immunological response resulting from the production of proinflammatory cytokines such as interferon-g, tumor necrosis factor-a, interleukin-1, interleukin-2, interleukin-6, and prostaglandin E2 (Hallab & Jacobs, 2017; Hallab et al., 2001). The types of metals that can induce hypersensitivity reactions include chromium, cobalt, nickel, platinum, mercury, titanium, silver, and gold (Zhang et al., 2016). They are commonly found in orthopedic devices such as surgical implants and joint prostheses (Innes & Atwater, 2020; Ramos et al., 2015), dental implants, fillings, and crowns (Basko-Plluska et al., 2011), and coronary devices such as stents (Meireles et al., 2007; Svedman et al., 2009). Benson et al. (1975) reported high rates of hypersensitivity to metals in orthopedic patients while Innes and Atwater (2020) highlighted the prevalence of metal hypersensitivity in dental patients.

Despite significant improvements in the quality of life for patients, many complications are associated with procedures involving metal-bearing devices (Eliaz, 2019). Localized effects include aseptic osteolysis in the periprosthetic area and loosening of the prosthesis, resulting in device failure (Hallab et al., 2001). Metal ions can also trigger a systemic immune response when they are released during corrosion and act as haptens, forming complexes with blood proteins (Anzengruber et al., 2019; Basko-Plluska et al., 2011). The clinical presentation of systemic metal hypersensitivity can include cutaneous and noncutaneous symptoms such as hyperemia, acute and chronic pain, dermatitis, eczema, urticaria, device failure, joint effusion, unexplained skin rashes, and delayed healing (Anzengruber et al., 2019; Basko-Plluska et al., 2011; Hallab & Jacobs, 2017; Hallab et al., 2001; Lhotka et al., 1998; Schalock & Thyssen, 2013).

These complications are not systematically evaluated because of gaps in the clinical investigations involving metal implants. Currently, regulatory bodies regulate the effectiveness of devices to address the clinical issues but not the material used in making them (Johnson, 2016). In the USA, many biomedical devices with metallic composition arrive on the market via the 510(k) clearance mechanism, which does not require randomized control clinical trials (Souza et al., 2019). This gap appears to suggest when investigating the effectiveness of implantable medical devices, mixed method research designs (e.g., simultaneous triangulation design (QUAN + qual) or sequential triangulation design (QUAN → qual) might be a better approach to elucidate safety concerns that may arise.

Metal hypersensitivity reactions following device implantation occur more frequently than previously thought. A study conducted in a dermatology and allergy/immunology clinic in Germany found that among 25 patients, 20 had a positive diagnosis for hypersensitivity to the metal used in knee arthroplasty based on the skin patch test (Thomas et al., 2009). This high rate of metal hypersensitivity was corroborated by another study showing that 20% to 30% of the world population has some form of hypersensitivity, with orthopedic surgery being one of the major causes of metal hypersensitivity (World Allergy Organization, 2013). Thus, it is important to investigate evidence-based surgical procedures involving metals and their associated adverse events to seek preventive measures for patients undergoing these procedures (Eliaz, 2019).

The use of metals and hypersensitivity reactions to metals are mostly associated with clinical interventions occurring in hospital or dental settings, where nurses play an integral role in the management of patients undergoing procedures requiring implantable devices. They contribute critical assessments (e.g., allergy assessment and patient education) that drive medical decision-making throughout the pre-, intra-, and postoperative phases. However, there is evidence that nurses lack implicit and explicit knowledge of hypersensitivity reactions to metal components in implantable devices (Dordunoo et al., 2021). How this evidence to practice gap in nursing affects patient outcomes is unknown but may contribute to the underreporting of metal hypersensitivity.

The gaps in knowledge regarding hypersensitivity reactions to metal have led to the lack of a consensus on preoperative screening or postoperative surveillance (Guimarães & Bezerra, 2019; Teo & Schalock, 2017; Xie et al., 2020). To facilitate a deeper knowledge of metal hypersensitivity, we undertook a bibliometric analysis to assess peer-reviewed articles to quantify explicit knowledge about metal hypersensitivity and identify gaps requiring further investigation (Alnajem et al., 2021; Duarte et al., 2005; Guimarães & Bezerra, 2019; Xie et al., 2020). In addition, although nurses may have tacit knowledge regarding this issue, it is missing from their training curriculum. By making knowledge of metal hypersensitivity explicit, we hope to propel innovation toward addressing this issue in the health systems.

## Methods

This bibliometric analysis first described the patterns of publications on the topic of metal hypersensitivity. A quantitative analysis was conducted based on descriptive analysis. It identified the most prolific authors, journals that frequently publish on the topic, and commonly used keywords. This type of study is increasingly applied to initiate technical activities and scientific research (Miguel et al., 2016; Xie et al., 2020).

This review used secondary data that are freely available in a public data repository, Scopus, thus it was exempted from ethics approval. We selected Scopus because it is an up-to-date and comprehensive database of publications with worldwide coverage in areas such as science, technology, medicine, social sciences, arts, and humanities (Elsevier, 2022). A single database search of Scopus was conducted in August 2020, with individual and connected (with AND) descriptors such as “metal," “allergy,” and “hypersensitivity.” As eligibility criteria, all articles had to be available online and indexed in English, Spanish, or Portuguese. The articles were assessed for inclusion and areas of conflict were resolved. Articles were excluded if they were duplicative or theses. We did not limit the search to given publication years because we wanted to retrieve all publications on the topic. The quality of the retrieved articles was not assessed because bibliometric analysis aims to map out publications without emphasis on their internal validity.

The Bibliometrix and Biblioshiny data packages in RStudio® (ver. 3.6.1) were used to analyze the following variables: total number of publications/year, publication country, institutions, journal, author name, and keywords. In VOSviewer® (ver. 1.6.6), we assessed the network of the most used descriptors among the retrieved articles (Guimarães & Bezerra, 2019) and analyzed the descriptor cooccurrence collaboration network. In addition, we used the bibliometric analyses tool on the Scopus platform.

## Results

**Figure 1.**
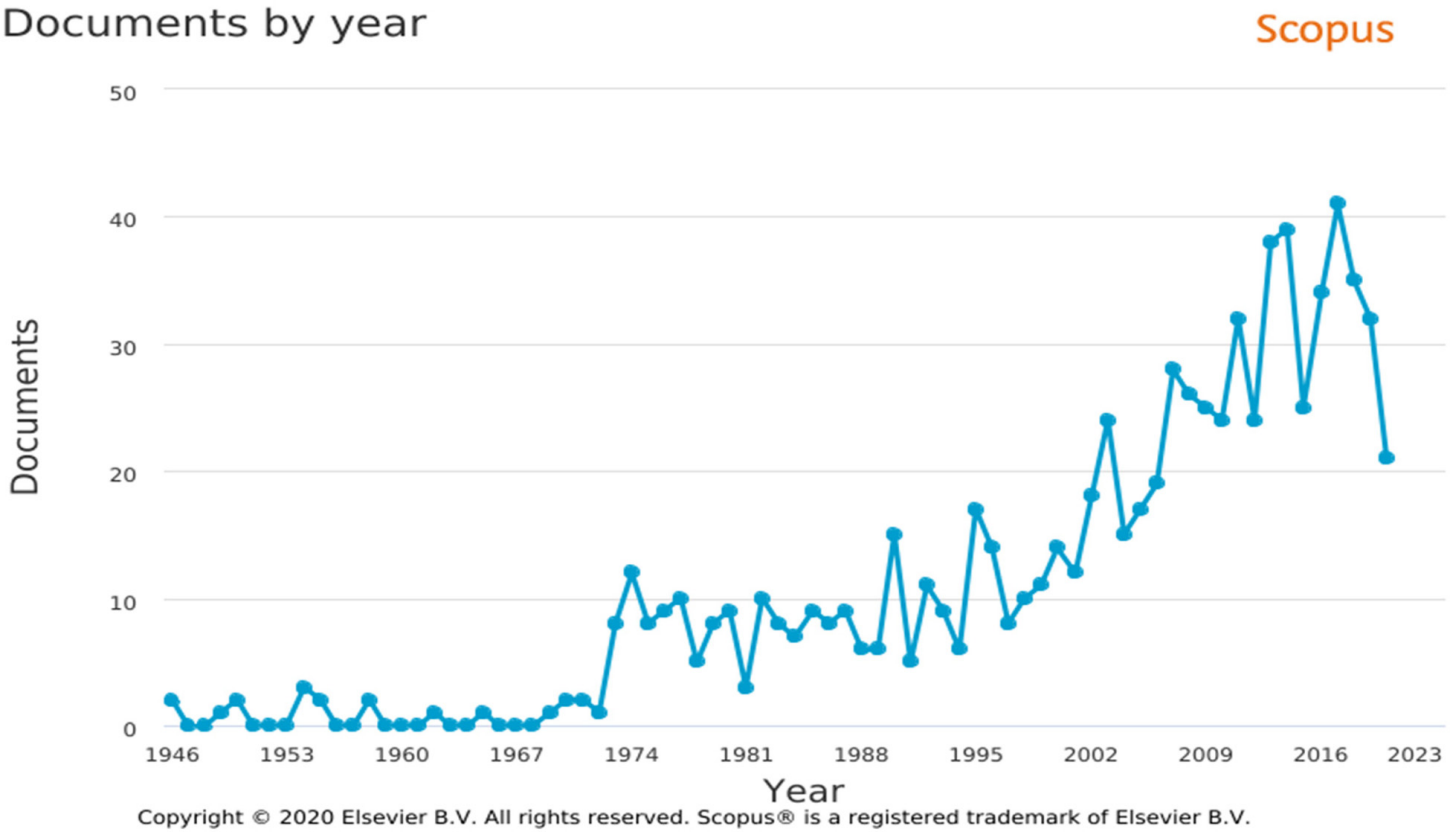
Annual evolution of scientific production on hypersensitivity.

*Source*: SCOPUS.

A total of 804 articles on metal hypersensitivity published between 1946 and 2020 were selected and included in the analysis (Figure 1). Interest in generating evidence about metal hypersensitivity increased with time, particularly after 1974. A mean of 18.5 articles/year was published on the subject from 1946 to 1974. The number gradually increased between 1974 and 2009. The mean annual number of articles for 2009–2016 was 30.1 compared to 32.6 for 2016–2020.

### Global Publishing Patterns

**Figure 2.**
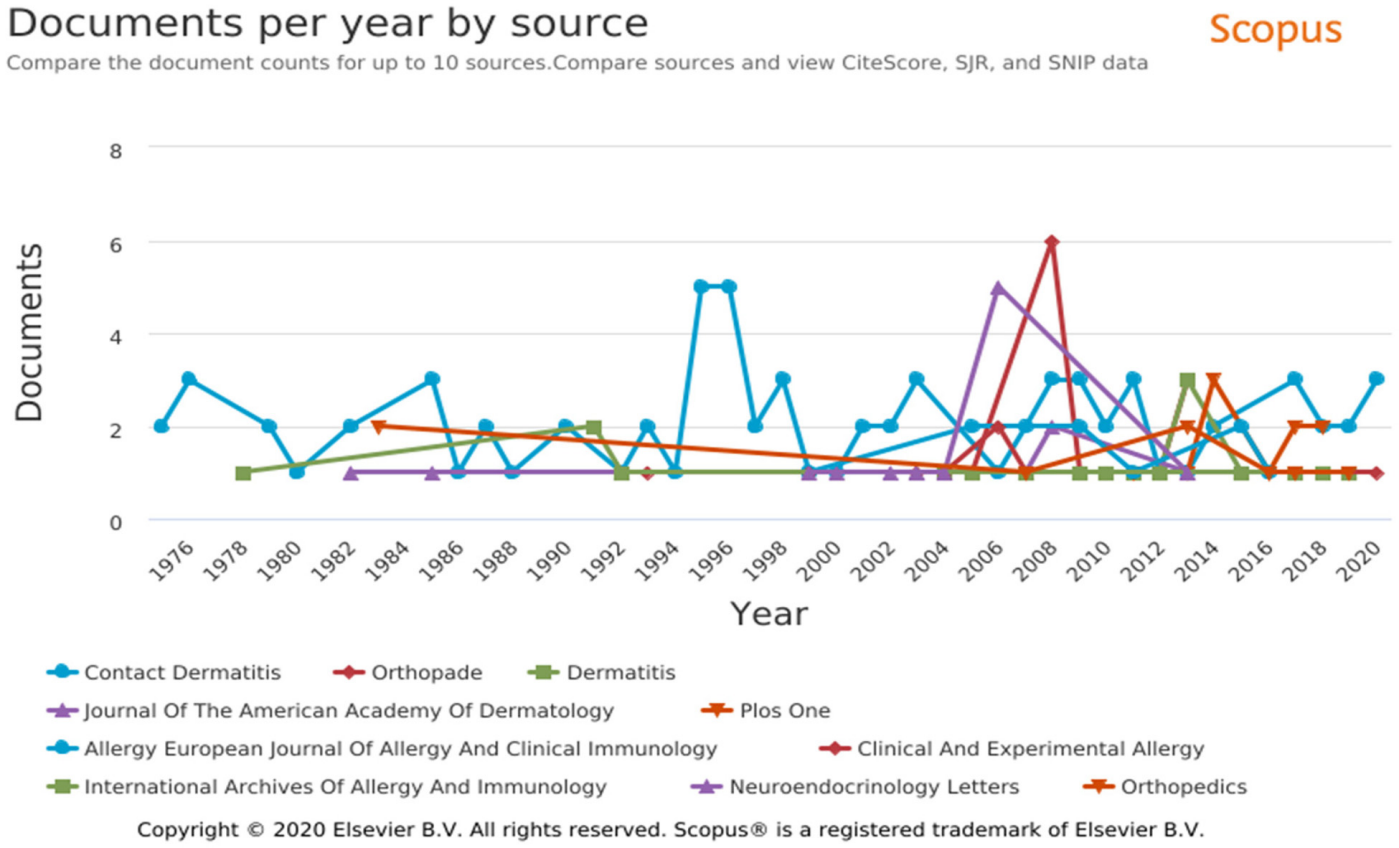
List of most cited journals per year of publication.

**Figure 3.**
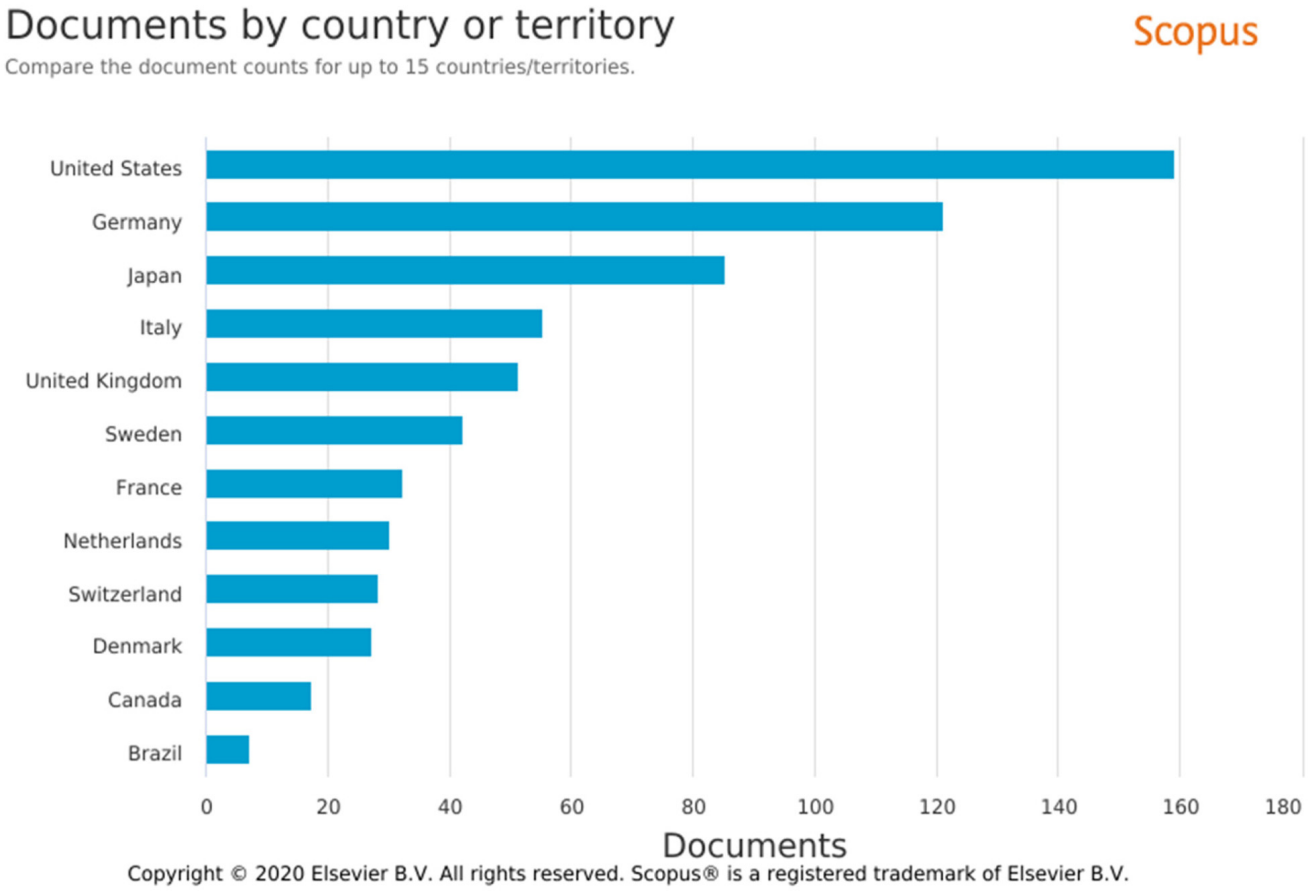
The 12 countries with the most publications on hypersensitivity. *Source*: SCOPUS.

The USA is the global leader with respect to the number of publications (*n* = 158, 19.6%), followed by Germany (*n* = 120, 14.9%), and Japan (*n* = 83, 10.3%). Only seven articles (0.87%) were published in Brazil, with one publication in each of 1993, 1995, 2008, 2010, 2013, 2016, and 2017 (Figures 2 and 3).

### Journals

Most articles (*n* = 75, 9.3%) were published in the journal, *Contact Dermatitis*, followed by *Orthopade* (*n* = 13, 1.61%) and *Dermatitis* (*n* = 12, 1.49%) (Figure 2). Publications in *Contact Dermatitis* were concentrated in 1995 (*n* = 5, 0.62%) and 1996 (*n* = 5, 0.62%), whereas the publication peaks were in 2008 in the *Journal of the American Academy of Dermatology* (*n* = 2, 0.24%) and *Orthopade* (*n* = 6, 0.74%).

### Institutional Affiliation

**Figure 4.**
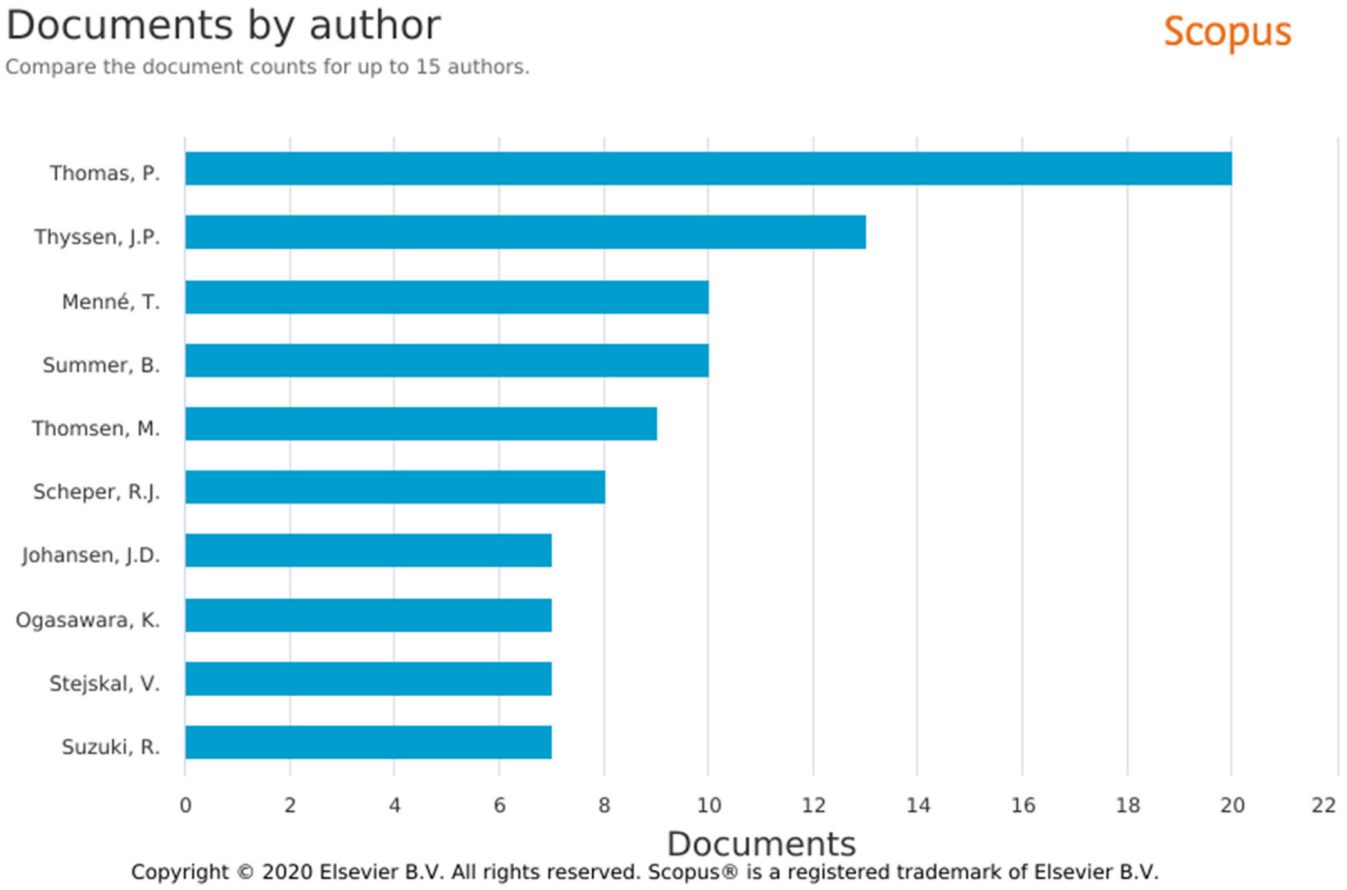
Number of articles published by author. *Source*: SCOPUS.

The most prolific authors were located at institutions in Germany (Figure 4): the Ludwig-Maximilians-Universitãt Munchen (*n* = 25, 3.10%) and Klinikum der Universitãt Munchen (*n* = 22, 2.73%). Although the USA had the highest total number of publications, the leading American institution, Harvard Medical School (*n* = 10, 1.24%), was ranked fifth for publishing on metal hypersensitivity.

### Authors

**Figure 5.**
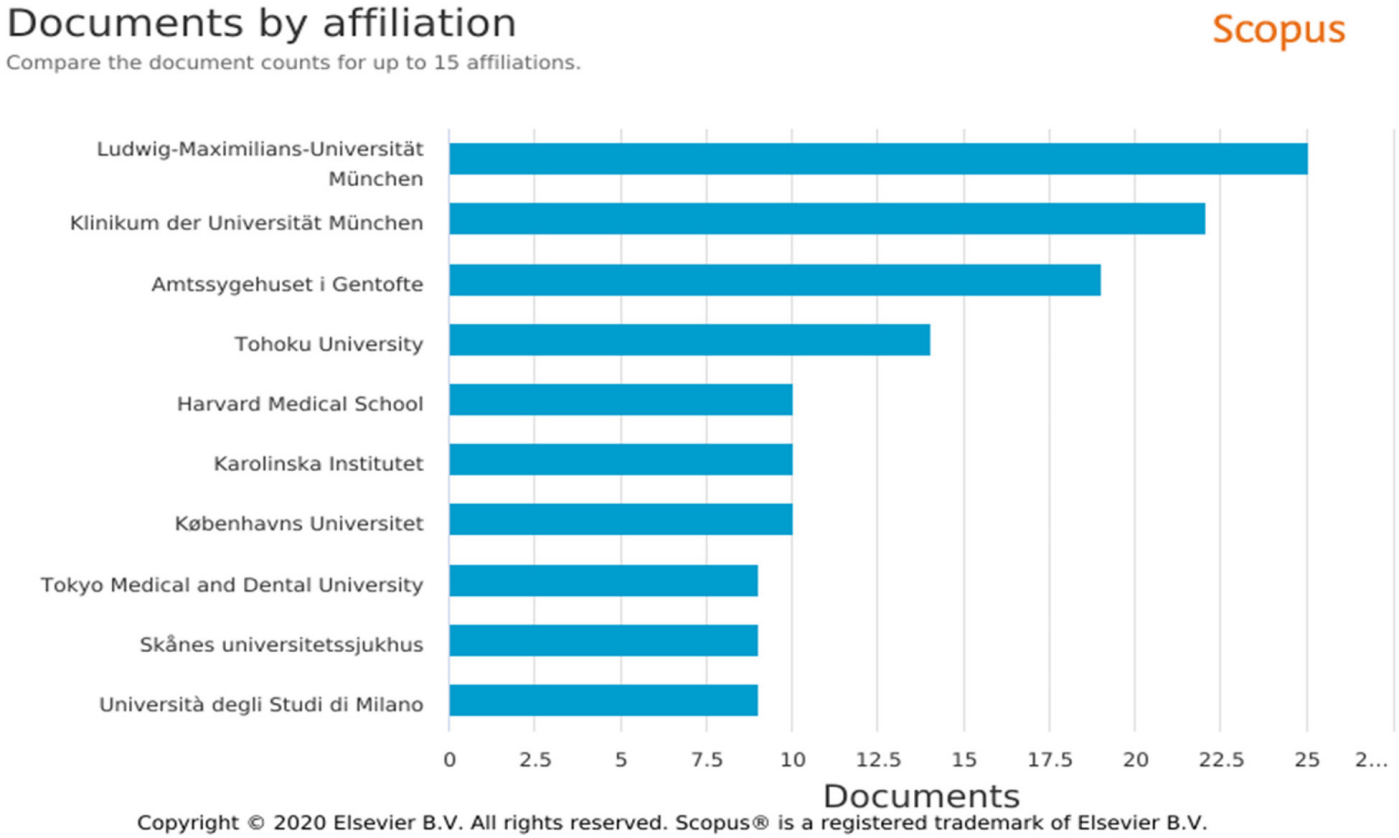
Number of publications per affiliations/institutions. *Source*: SCOPUS.

Few authors had more than six articles on metal hypersensitivity (Figure 5). Among the 10 authors who published the most on the topic, Peter Thomas had 20 (2.48%) articles, five of which were published in 2008. Jacob Thyssen authored 13 (1.61%) articles, followed by T. Menné (*n* = 10, 1.24%) and B. Summer (*n* = 10, 1.24%).

### Keywords

**Figure 6.**
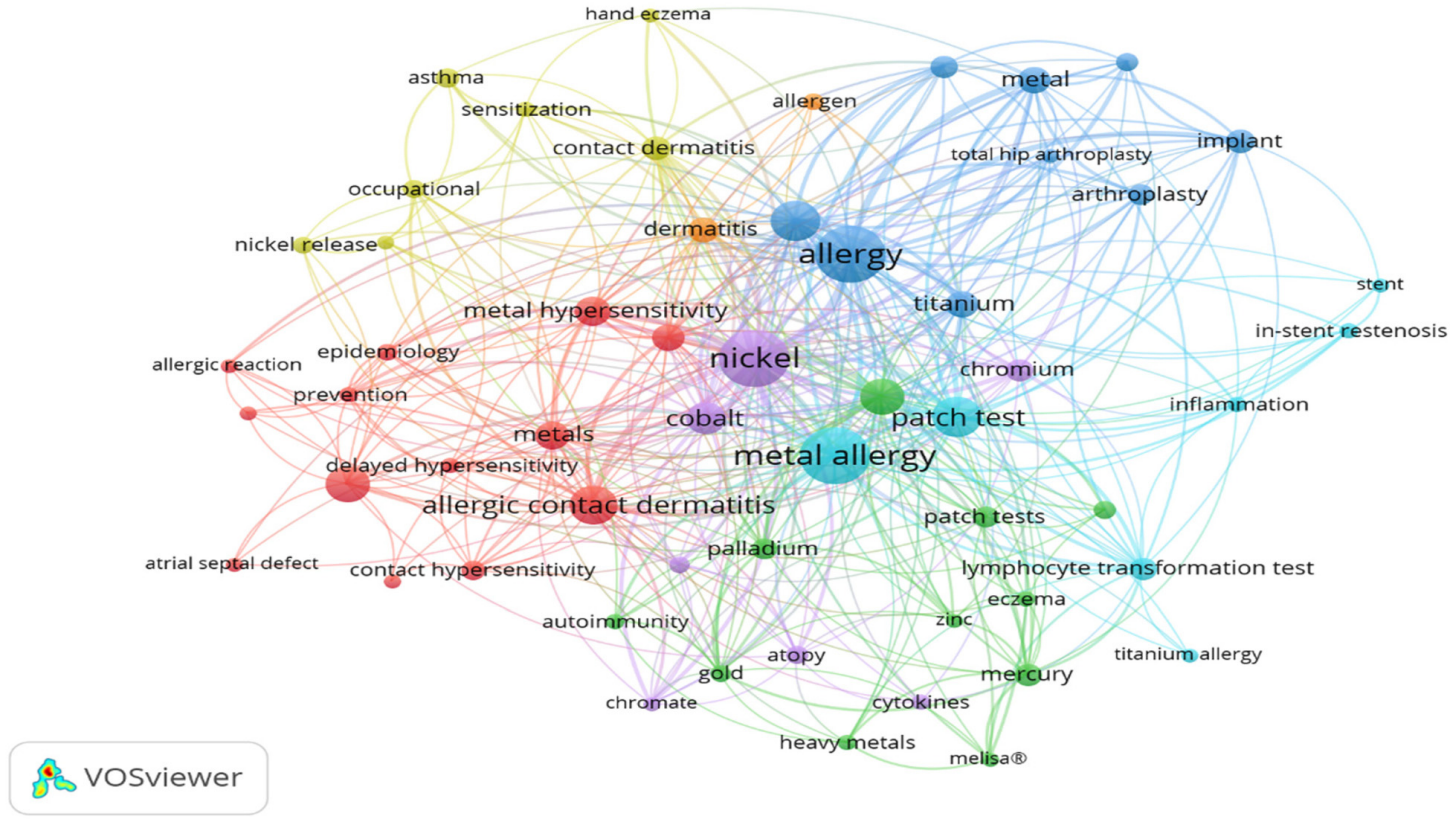
Cooccurrence of keywords among articles. *Source*: Own elaboration.

**Figure 7.**
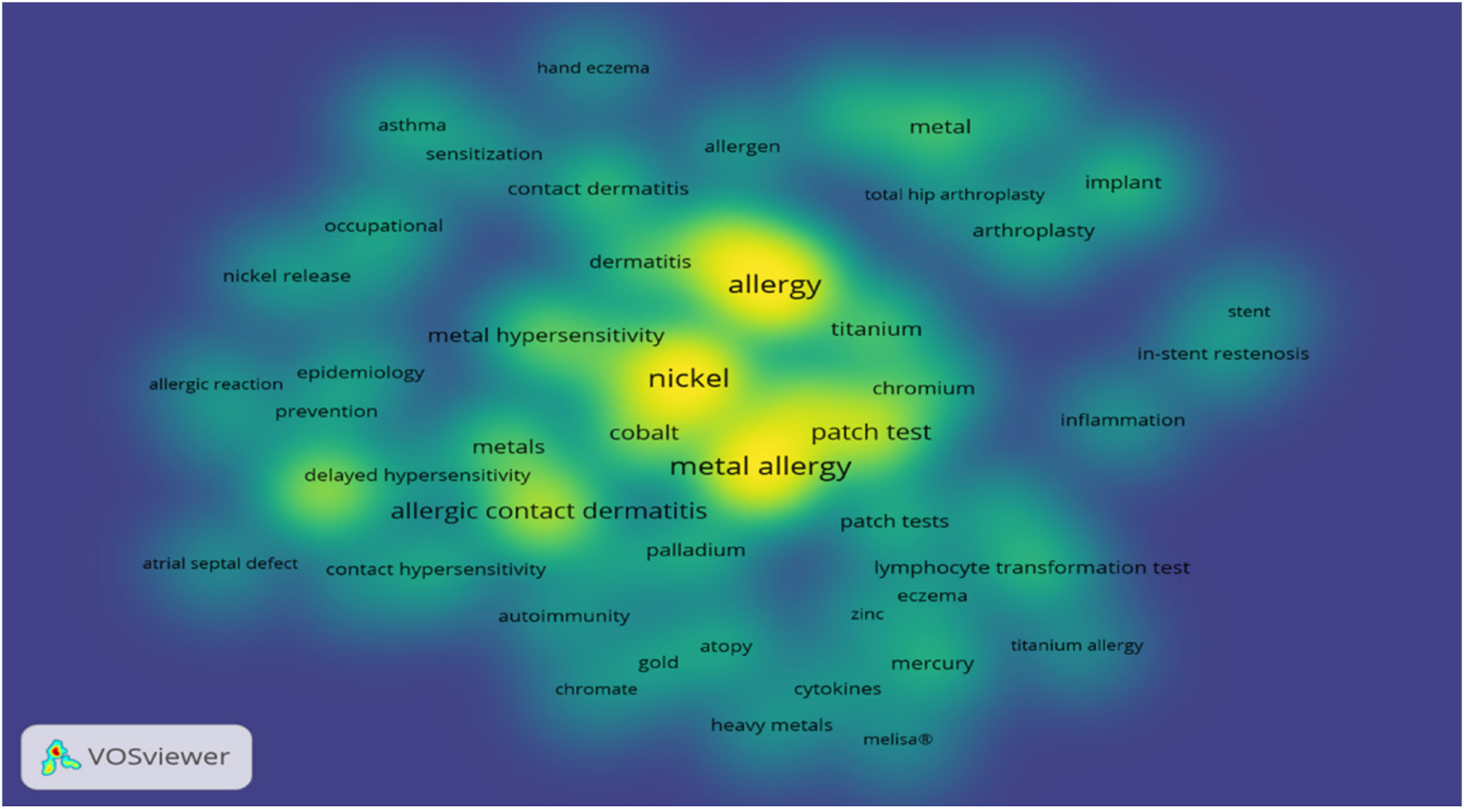
Cooccurrence of keywords related to color intensity. *Source*: Own elaboration.

The visualization of keyword formation through a network showed a total of 1,368 keywords in the 804 articles (Figure 6). We connected cooccurrences of keywords in a set of seven colors. Red, blue, and green are the dominant colors, suggesting that most publications were on the topics of research methodologies (red), followed by types of metals and surgeries (blue), and diagnostic measures (green). The node size indicates the frequency of occurrence, and the curves between nodes represent their cooccurrence in the same publication. The shorter the distance between two nodes, the larger the number of cooccurrences of the two keywords. The descriptor analysis (Figure 7) depicts the keyword distribution in the articles, correlating their density with the frequency of descriptor cooccurrences. The most frequent descriptors are yellow, among which four had the greatest impact: allergy, metal allergy, nickel, and patch test.

## Discussion

This study found a gradual increase in the number of publications on metal hypersensitivity in recent years, with the highest number of articles published between 2016 and 2020. This growth has also been observed in other knowledge areas and may be associated with more research funding and incentives worldwide (Peters, 2019). In addition, technological investments resulted in the expansion of experimental studies and surgical procedures involving metal use (Bond et al., 2019). For example, surgeries involving implants with metal compositions increased globally from 1.7 million in 2012 to 2.9 million in 2016. More than 5 million metal implant surgeries were projected to have occurred in 2021 (Murr, 2020). A study published by the University of Milan, Italy, revealed an increase in the number of orthopedic implants with metal compositions in recent decades, mainly in hip and knee prostheses (Sansone et al., 2013).

The USA and Germany are the most responsible for the significant increase in the number of publications on metal hypersensitivity since 2010. Universities are the main institutions advancing science. Approximately 31.5% of well-known research universities around the world are in the USA, which directly contributes to new innovations in technology and novel approaches to modern-day scientific dilemmas more efficiently (Peters, 2019; Ximenes et al., 2019). Although the USA leads in the absolute number of publications, we found that the most active institutions are concentrated in Germany. Germany has large university centers that focus resources on scientific research, whereas, in the USA, resources appear to be distributed among several universities (Peters, 2019; Ximenes et al., 2019).

However, investment in research has declined over the years in countries such as Brazil, which is reflected in the quantity and quality of scientific publications (Conti, 2020). Low investment in research and innovation left many of these countries unprepared to face the severe-acute respiratory syndrome-coronavirus-2 pandemic (Ministério Da Educação, Portugal, 2020). Research forms the basis for the development of new medicines, instruments, and technological equipment. Therefore, investment cuts in research contribute to dependence on international knowledge and technology to improve the health of its citizens (Conti, 2020).

This endeavor will also support research in other disciplines to find alternate materials for implantable devices. Funding agencies and universities also need to support studies in this area to find reliable in vitro assays and other biomarkers that can enhance the patient selection process and the clinical management of those who develop hypersensitivity reactions. Since the research and innovation processes are closely linked to publication, funding is a necessary condition for the advancement of knowledge dissemination and scientific production.

The journals that publish more on metal hypersensitivity were in the field of orthopedics and dermatology. This suggests that, whereas metals are heavily used in orthopedics, dermatology serves as the consulting diagnostic service. Nurses are the most proximal clinician to the patients; however, we identified a lack of publications in nursing journals.

The leading author on metal hypersensitivity, Peter Thomas, focuses on orthopedic surgery, while Jacob Thyssen has expertise in dermatology. Only one study was conducted by nurse researchers, which contributes to the lack of explicit knowledge among nurses. Screening for hypersensitivities in clinical practice is an activity that nurses perform routinely, thus educating them about metal hypersensitivity can help identify gaps in clinical practice, reduce risks to patient health, and increase the success of the implant (Dordunoo et al., 2021; Kumar et al., 2016). We emphasize the importance of expanding investments in nursing research to enhance the detection and clinical management of metal hypersensitivity. More journals, particularly those targeting nurses, need to publish research about metal hypersensitivity to increase the reach of the evidence.

Among the seven sets of keywords, the most cited by the authors were “hypersensitivity,” “metal hypersensitivity,” and “nickel.” These descriptors support the finding in the literature that nickel is the leading cause of metal hypersensitivity worldwide (Ahlström et al., 2019). Among medical records containing data from skin patch tests, 59.2% of the patients had at least one positive hypersensitivity to some metal, and nickel was the most common metal to induce hypersensitivity reactions (Rodrigues & Goulart, 2015). This is further supported by our study, which shows the metals that were most cited were nickel and cobalt. Identification of patients with a history of metal hypersensitivity reactions is required to reduce the risk of complications and adverse events associated with interventions. However, this topic is still poorly studied/explored.

More investment is needed in studies to improve the knowledge of professionals, patients, and researchers regarding hypersensitivity to metals as well as their risks, diagnostic tests, and prognosis following implant (Sansone et al., 2013). Metal hypersensitivity is a multifaceted problem that presents an opportunity to apply translational and multidisciplinary research teams and approaches. Translational research is delineated into three phases that aim to expedite the movement of evidence from basic research, to patient-oriented research, and to population-based research with the long-term goal of improving public health (Rubio et al., 2010). Expediting evidence transfer from basic science (bench) to the clinical arena (bedside) requires the engagement of researchers, healthcare providers, patients, and policymakers. Nurses occupy positions in each of these sectors of society and can play vital roles in moving evidence into practice and care coordination.

### Limitations

The use of bibliometric indicators is necessary and extremely valuable for the creation of strategic actions related to the evaluation and qualification of scientific production on a given topic. We emphasize that hypersensitivity reactions to metals are a complex issue that is far from being fully explored. Thus, our aim was to provide a critical view of scientific publications on metal hypersensitivity. The use of a single database restricted the number of articles retrieved on the subject, although this database is considered a multidisciplinary database with extensive coverage of the medical area.

### Implications for Practice

Nurses are at the forefront of patient care; however, evidence suggests that they are unaware of hypersensitivity reactions to metals in implantable devices. As a result, they do not ask patients about this when assessing them preoperatively. The findings of this review indicate a high variation in publishing rates from 1946 to 2020. Most publications are from the USA, Germany, and Japan but in journals and disciplines other than nursing. This may be contributing to the lack of awareness among nurses about this issue. The findings also indicate few nurses are involved in the research in this area, highlighting a need to increase the dissemination of the evidence among healthcare professionals. Knowledge translation in nursing about this issue can help inform future research into screening practices to support the detection of metal hypersensitivity.

## Conclusions

Scientific publications on metal hypersensitivity are relatively scarce but are increasing. The USA, Germany, and Japan appear to invest the most in scientific and technological developments in this field. The main scientific journals that publish on the topic are related to orthopedic procedures in which metals are used most frequently.

The scientific publications assessed here highlight the importance of disseminating knowledge about metal hypersensitivity to health professionals, who need to recognize the risks and adverse reactions from the use of metals in surgical procedures. Policies must be developed and implemented to screen for metal hypersensitivity, and future studies are needed to investigate the benefits of screening for metal hypersensitivity before and after a procedure.
